# Hesperidin alleviates hypothyroidism-related cardiac dysfunction by targeting cardiac miRNAs, Nrf2/NF-κB signaling, oxidative stress and inflammation

**DOI:** 10.3389/fphar.2025.1553992

**Published:** 2025-07-02

**Authors:** Asmaa M. Gaber, Adel Abdel-Moneim, Eman S. Abdel-Reheim, Gamal Allam, Manal Abdul-Hamid, Ahmed Hosni

**Affiliations:** ^1^ Molecular Physiology Division, Department of Zoology, Faculty of Science, Beni-Suef University, Beni-Suef, Egypt; ^2^ Immunology Division, Department of Zoology, Faculty of Science, Beni-Suef University, Beni-Suef, Egypt; ^3^ Histology and Cell Biology Division, Department of Zoology, Faculty of Science, Beni-Suef University, Beni-Suef, Egypt; ^4^ Department of Medical Biochemistry and Cell Biology, Institute of Biomedicine, University of Gothenburg, Gothenburg, Sweden

**Keywords:** hypothyroidism, cardiac dysfunction, hesperidin, miRNAs, oxidative stress, inflammation

## Abstract

**Background:**

Hypothyroidism is a frequent endocrine health issue that is linked to adverse cardiovascular events. Accumulating evidence suggests that thyroid hormone replacement does not fully reverse the cardiovascular complications associated with the disease despite normalization of serum thyroid hormone levels, indicating a need for adjunctive, complementary, or alternative therapies. Hesperidin (HSD) has diverse pharmacological activities, however, its therapeutic potential on the crosstalk between hypothyroidism and cardiac dysfunction has not been previously reported.

**Methods:**

This study aimed to investigate the cardioprotective efficacy of HSD on carbimazole (CMZ)-induced hypothyroidism in rats in comparison to the traditional thyroid hormone replacement therapy; levothyroxine (LT4). Male Wistar albino rats were divided into four groups: normal control (NC), CMZ (30 mg/kg), CMZ + HSD (30 mg/kg CMZ + 200 mg/kg HSD), and CMZ + LT4 (30 mg/kg CMZ + 0.045 mg/kg). All doses were given orally and daily for 9 weeks.

**Results:**

CMZ intake resulted in a significant decrease in thyroid hormones (THs) levels with a subsequent increase in serum thyroid stimulating hormone and cardiac enzymes activities, dyslipidemia, and body weight gain. Cardiac tissues revealed marked oxidative stress, inflammation, and structural degenerative lesions. As well, cardiac expression of miRNAs-92a and -499 was elevated while that of miRNA-21 was depleted, reflecting an interdependence between hypothyroidism and the development of cardiac dysfunction. Despite HSD and LT4 effectively alleviating the THs profile, only HSD offered substantial protection from hypothyroidism-associated cardiac inflammation and injury through its potent impact on the transcriptional miRNAs level and Nrf2/NF-κB protein expression (key regulators of the redox biomarkers and the inflammatory mediators).

**Conclusion:**

HSD provides dual thyroprotective and cardioprotective effects that enhance THs bioavailability and functionality in the cardiovascular system.

## 1 Introduction

Thyroid dysfunction is a common endocrine disorder that affects all populations worldwide and causes severe health consequences. The most prevalent thyroid abnormalities include hypothyroidism, hyperthyroidism, thyroid cancer, and Hashimoto’s thyroiditis. Hypothyroidism is the condition of thyroid hormones (THs) deficient secretion that affects more than 10% of the global population (Das et al., 2022).

As the thyroid hormones receptor (TR) is expressed in the myocardium and the vasculature, any alterations of THs level could have significant effects on cardiovascular functions by initiating different genomic and non-genomic actions (Jabbar et al., 2017; Zhang et al., 2017). The genomic actions of THs are mediated through the binding to the TR (a ligand-dependent transcription factor that belongs to the nuclear receptor superfamily) that attaches to the thyroid hormone response element, affecting the expression of the target genes through heterodimerization with the retinoid X receptor (RXR) (Zhang et al., 2017). Many clinical studies have shown that hypothyroidism can lead to hypertension, dyslipidemia, and various cardiovascular diseases (CVDs) (Delitala et al., 2017; Jabbar et al., 2017). Hypothyroidism can cause cardiac dysfunction by disrupting the cardiomyocytes’ Ca^2+^ homeostasis that impairs mitochondrial functions, resulting in excessive reactive oxygen species (ROS) generation, contractile failure, and cardiac tissue injury (Jabbar et al., 2017; Yamakawa et al., 2021). As well, the prolonged shortage of THs triggers the release of multiple inflammatory cytokines (Lai et al., 2023) that aggravate oxidative stress and mediate cardiac inflammation, leading to the progression of CVDs in hypothyroid patients (Balamurugan et al., 2023). As thyroid hormone replacement does not address oxidative stress, inflammation, or myocardial apoptosis, targeting these factors could be a promising therapeutic application in the prevention and management of hypothyroidism-related CVDs.

Non-coding RNAs are a huge family of genomic RNA that maintains essential biological features within the cells. MicroRNAs (miRNAs) are among the most extensively studied and best characterized non-coding RNAs (Gonzalez-Candia et al., 2024). MiRNAs can directly attach to target genes and influence their expression. Consequently, miRNAs were reported as biomarkers for several pathogenic conditions and recently appointed as the next-generation therapeutic targets (Hanna et al., 2019). Increasing evidence suggests that miRNAs play a vital role in the onset and development of CVDs (Gonzalez-Candia et al., 2024), with a lot of curiosity toward miRNAs-92a, −499, and −21 which are influenced by the thyroid dysregulation, suggesting an interplay between THs-signaling and progression of cardiac dysfunction (van Rooij et al., 2009; Zhang et al., 2017; Aranda, 2021). Therefore, studying the efficacy of potential therapeutics on modulating cardiac miRNAs will provide a novel strategy to protect from hypothyroidism-related cardiac dysfunction and a step forward to develop miRNAs-related drugs.

Levothyroxine (LT4; an exogenous synthetic thyroid hormone) has long been used as the standard drug for the treatment of hypothyroidism (Chiovato et al., 2019). However, this traditional tablet formulation failed to achieve the recommended serum level of the thyroid stimulating hormone (TSH) and caused cardiovascular, neuropsychiatric, gastrointestinal, dermatological, and endocrine adverse effects with incorrect dosing (Bousquet et al., 2005; Gluvic et al., 2021; Nagy et al., 2021). As well, some comorbidities influence LT4 bioavailability and affect its optimal action. Therefore, its ingestion necessitates excellent patient compliance and frequent dose adjustments which are challenging with the longstanding treatment.

There is a substantial motivation to use natural products from medicinal plants as curative agents for various diseases. Hesperidin (HSD) is a major dietary flavanone that is present in high concentrations in many citrus fruits in peels, pulp, seeds, and membrane residues (Welbat et al., 2020; Samota et al., 2023). Recently, Samota and colleagues reviewed HSD therapeutic benefits and highlighted its antitumor, antibacterial, anti-inflammatory, antidiabetic, antioxidant, and hypolipidemic properties (Samota et al., 2023). As well, it has attracted increasing interest in treating cardiovascular complications (Khorasanian et al., 2023). However, the therapeutic potential of HSD on hypothyroidism-related cardiac dysfunction has not yet been studied. Carbimazole (CMZ) is an antithyroid drug that is commonly used as an induction agent for experimental hypothyroidism. After ingestion, it is immediately metabolized into methimazole (MMI). MMI inhibits the thyroid peroxidase action, decreasing the incorporation of iodide into tyrosine residues on the thyroglobulin and leading to a reduction in thyroid hormone synthesis (Abbara et al., 2020). In this study, we aimed to explore the cardioprotective efficacy of HSD on CMZ-induced hypothyroidism in rats and to understand its mechanistic action using targeted molecular, biochemical, and histopathological analysis compared to LT4 as a reference medication.

## 2 Material and methods

### 2.1 Chemicals and drugs

Carbimazole, as the hypothyroidism-stimulating agent, was purchased from Chemical Industries Development (CID) Company, Egypt. We bought hesperidin from Merk (Sigma-Aldrich^®^ Brand), and levothyroxine^®^50mcg from GlaxoSmithKline NZ, Germany. The remaining chemicals were procured from standard commercial sources.

### 2.2 Experimental animals

Male Wistar albino rats (*Rattus norvegicus*) of body weight 120 ± 10 g were acquired from Nahda University (the Animal House Facility), Beni-Suef, Egypt. They were kept under normal conditions of the light/dark cycle, temperature, and humidity. Rats were given free access to standard rat chow and water during the experimental period. Considerable efforts were made to reduce the animals’ suffering and the numbers included. All animal experiments were approved by the Institutional Animal Care and Use Committee of Beni-Suef University, Egypt (IACUC-BSU, no. 019-76) which follows the guidelines of the National Institute of Health.

### 2.3 Experimental design

A total of 24 male Wistar albino rats were divided randomly into four groups (six/group) as follows:

⁃ Normal control rats (NC): given distilled water.
⁃ Carbimazole induced hypothyroid rats (CMZ): given carbimazole (30 mg/kg b.wt. (Abdel-Moneim et al., 2015) dissolved in distilled water).
⁃ Hypothyroid rats with hesperidin treatment (CMZ + HSD): given hesperidin (200 mg/kg b.wt. (Varisli et al., 2022) dissolved in 1% carboxymethyl cellulose) in concomitant to CMZ dosing.
⁃ Hypothyroid rats with levothyroxine treatment (CMZ + LT4): given levothyroxine (0.045 mg/kg b.wt. (Paget and Barnes, 1964) dissolved in distilled water) in concomitant to CMZ dosing. All doses were given orally and daily (between 8.00-10.00 a.m.) for 9 weeks.

At the end of the experiment, all rats were fasted for 12 h and sacrificed under diethyl ether anesthesia. Blood was collected, and sera were separated and kept at −24°C for analysis. Fresh heart tissue samples were preserved in RNA *later* ^®^ (Merk., Germany) at −80°C for the gene expression analysis. Other samples were stored at −80°C for the protein expression analysis and biochemical assays. As well, cardiac tissue samples were fixed in neutral buffered formalin (NBF; 10%) for paraffin-section preparation and histopathological screening, while others were fixed in glutaraldehyde (3%) at 4°C for ultrastructural examination.

### 2.4 Biochemical measurements

#### 2.4.1 Serum thyroid hormones

ELISA kits from MyBioSource Inc., USA, were used to quantitatively measure the serum levels of total T3, total T4, free T3, free T4, and TSH (SKU no. MBS580039, MBS580037, MBS704457, MBS700784, and MBS701641, respectively) following the manufacturer’s instructions.

#### 2.4.2 Cardiac function enzymes activities and proteins levels

Serum activity of total creatine kinase (CKT), creatine kinase-MB (CK-MB), and aspartate aminotransferase (AST) was detected using kits purchased from Biodiagnostic Co., Egypt. Cardiac content of Troponin-T and -I was measured using ELISA kits from MyBioSource, Inc., USA (SKU no. MBS2703747 and MBS727624, respectively).

#### 2.4.3 Serum lipid profile and cardiovascular risk (CVR)-indices

The serum triglycerides (TG), total cholesterol (total Ch.), and HDL-cholesterol (HDL-Ch.) levels were estimated using colorimetric kits from Bio-diagnostic Co., Egypt, according to the manufacturer’s protocols. The serum LDL-cholesterol (LDL-Ch.) level was calculated following Friedewald’s formula (Friedewald et al., 1972): LDL-Ch. = Total Ch. – (TG/5 – HDL-Ch.), while the vLDL-cholesterol (vLDL-Ch.) was estimated using Norbert’s equation (Norbert, 1995): vLDL-Ch. = TG/5. The CVR indices were determined using the following formulas (Ross, 1993): CVR1 = Total Ch./HDL-Ch., and CVR2 = LDL-Ch./HDL-Ch. The anti-atherogenic index (AAI) was computed using this equation (Guido and Joseph, 1992): AAI = (HDL-Ch. ×100)/(Total Ch. – HDL-Ch.).

#### 2.4.4 Cardiac redox status and inflammatory mediators

Malondialdehyde (MDA; as a lipid peroxidation biomarker), and nitrite (as the nitric oxide “NO” indicator) levels were detected in the heart tissue homogenate according to methodologies by Ohkawa et al. (1979) and Montgomery and Dymock (1961), respectively. Cardiac content of glutathione (GSH) and enzyme activities of catalase (CAT) and superoxide dismutase (SOD), as antioxidant parameters, were determined using kits from Bio-diagnostic Co., Egypt, according to the guidelines.

The proinflammatory cytokines tumor necrosis factor alpha (TNF-α), interleukin (IL)-1β, IL-6, and IL-17 were quantified in the cardiomyocytes by ELISA kits from MyBioSource Inc., USA (SKU no. MBS700574, MBS825017, MBS2020158, and MBS2022678, respectively). The anti-inflammatory mediators IL-10, IL-37, and peroxisome proliferator activated receptor alpha (PPAR-α) were measured in the cardiac tissues using MyBioSource ELISA kits (SKU no. MBS764911, MBS8807693, and MBS2504779, respectively).

### 2.5 Molecular analysis

#### 2.5.1 Cardiac gene expression of miRNAs-92a, −499 and −21, NOS (endothelial and inducible), and caspase-3/BAX apoptotic markers

The real-time polymerase chain reaction was used to quantify the gene expression levels of miRNAs-92a, −499, and −21 (with U6 as an internal control), as well as the inducible nitric oxide synthase (iNOS), endothelial nitric oxide synthase (eNOS), caspase-3, and BCL2-associated X protein (BAX) (with β-actin as an internal control). The procedure was carried out in accordance with a standardized protocol (Pfaffl, 2001). Concisely, total RNA was extracted from the heart tissues using the TRIzol^TM^ reagent (Invitrogen, USA). The RT-PCR kit (SuperScript™ IV one-step; SKU no. 12594100, Thermo Scientific, USA) was used for the reverse transcription of the isolated RNA. The cDNA was amplified using SYBR-green master mix (Fermentas, USA), and the primers used are listed in Table 1 (Vivantis Technologies, Malaysia). The obtained data were analyzed using the 2^−ΔΔCT^ method (Livak and Schmittgen, 2001), where the values were normalized either to U6 or β-actin and expressed in percentage as compared to the control.

**TABLE 1:** List of primers.

Primer	Forward sequence (5′–3′)	Reverse sequence (5′–3′)
miRNA-499	5′-TTA​AGA​CTT​GCA​GTG​ATG​TTT-3′	5′-CAG​TGC​AGG​GTC​CGA​GGT​AT-3′
miRNA-92a	5′-ATA​ACG​TGA​ACA​GGG​CCG​G-3′	5′-CAG​TGC​GTG​TCG​TGG​AGT-3′
miRNA-21	5′-TAG​CTT​ATC​AGA​CTG​ATG​TTG​A-3′	5′-GAG​GTA​TTC​GCA​CTG​GAT​ACG-3′
U6	5′-CTC​GCT​TCG​GCA​GCA​CA-3′	5′-AAC​GCT​TCA​CGA​ATT​TGC​GT-3′
eNOS	5′-CGA​GAT​ATC​TTC​AGT​CCC​AAG​C-3′	5′-GTG​GAT​TTG​CTG​CTC​TCT​AGG-3′
iNOS	5′-CAC​CAC​CCT​CCT​TGT​TCA​AC-3′	5′-CAA​TCC​ACA​ACT​CGC​TCC​AA-3′
BAX	5′-CGG​CGA​ATT​GGA​GAT​GAA​CTG​G-3′	5′-CTA​GCA​AAG​TAG​AAG​AGG​GCA​ACC-3′
Caspase-3	5′-GTG​GAA​CTG​ACG​ATG​ATA​TGG​C-3′	5′-CGC​AAA​GTG​ACT​GGA​TGA​ACC-3′
β-actin	5′-AAG​ATC​CTG​ACC​GAG​CGT​GG-3′	5′-CAG​CAC​TGT​GTT​GGC​ATA​GAG​G-3′

#### 2.5.2 Western blotting of cardiac Nrf2 and NF-κB proteins

Heart tissue samples were homogenized in the ice-cold lysis buffer and centrifuged at 14,000 xg for 10 min at 4°C. The protein quantities were assessed by Bradford’s method (Bradford, 1976). Then, the samples were mixed properly with the loading buffer and heated for 8 min at 95°C. Proteins were separated by SDS/PAGE and transferred to the PVDF membranes. Blots were incubated in the blocking buffer (5% non-fat milk + 0.1% Tween 20 in Tris-buffered saline; TBS-T) for 2 h at room temperature (R.T.). The primary antibodies (1^ry^Abs) for the nuclear factor erythroid 2-related factor 2 (Nrf2), the nuclear factor kappa-B (NF-κB), and β-actin were prepared at 1:1000 dilution. The membranes were incubated with 1^ry^Abs overnight at 4°C, then washed in TBS-T three times for 15 min each. Lastly, blots were incubated with the corresponding 2^ry^Ab for 3 h at R.T., then washed as previously described. Protein bands were visualized using the chemiluminescent Pierce^TM^ ECL-Western blotting substrate and the Alliance 4.7 gel documentation (UK). The UV Tec software was used to semi-quantify the protein bands. Every band was calibrated using β-actin as the reference.

### 2.6 Histopathological, immunohistochemical and ultrastructural examinations

Heart tissue samples (*n =* 3/group) were fixed for 24 h in 10% NBF, dehydrated through a series of the ethyl alcohol, cleared with xylene, paraffin-embedded at 60°C, and then sectioned (4–5 μm thickness). Slides were either stained with hematoxylin and eosin (H&E) (Gamble, 2008) for the histopathological examination or immunostained for the detection of galectin-3 (Gal-3) and matrix metalloproteinase-9 (MMP-9) expression (Petrosyan et al., 2002) as markers of cardiac inflammation and fibrosis.

For the ultrastructural examination, cardiac tissue samples (*n =* 3/group) were fixed in 3% glutaraldehyde in phosphate buffer for 2 h. After washing, samples were post-fixed in isotonic osmium tetroxide (1%) and embedded in Epon 812 according to a standard procedure (Kishi-Itakura et al., 2014). Ultra-thin sections were stained in the uranyl acetate and lead citrate solutions and then examined by transmission electron microscopy (JEOL CX 100, Japan).

The scoring of histopathological and ultrastructural abnormalities was assessed from six random fields of each sample slide. The severity of lesions was classified into four grades: (−) absent, (−/+) few “≤ 10%”, (+) moderate, and (++) severe. Images were evaluated by two assessors who were blind to the related treatment. Further, the histochemical scoring (H-Score) was used to semi-quantify immunostaining of Gal-3 and MMP-9 (Ponglowhapan et al., 2008). Briefly, four levels were used to categorize the staining intensity: (0) for negative staining, (1) for low staining, (2) for moderate staining, and (3) for heavy staining. As well, the percentage of the positively stained cells was divided into five grades: (0) for < 5%, (1) for 6%–25%, (2) for 26%–50%, (3) for 51%–75%, and (4) for > 75%. The staining intensity and the percentage of positively stained cells were multiplied to show the final score.

### 2.7 Statistical analysis

The one-way ANOVA test followed by Tukey’s post-hoc analysis was made for all statistical comparisons using SPSS v.25.0 (Chicago, USA). Results were presented as means ± SEM. Significant effects were considered at *p* < 0.05.

## 3 Results

### 3.1 Effect of HSD and LT4 on the serum thyroid hormones level, the body weight changes and the cardiac function biomarkers of CMZ-induced hypothyroidism

Table 2 illustrates a significant decrease in the serum THs (total T3, total T4, free T3, and free T4) along with a marked increase in the TSH level of CMZ-induced hypothyroid rats as compared to the NC-group. Consequently, the body weight of hypothyroid rats was notably higher than the normal controls. This could be due to the slowdown of the metabolic processes secondary to THs deficiency (Lustig et al., 2022). The administration of HSD effectively alleviated the levels of THs and TSH up to the normal values better than LT4, while both comparably controlled the body weight gain. As the altered THs directly affect cardiac physiology (Yamakawa et al., 2021), our study revealed a potent increase in the serum cardiac enzymes activities (CK-MB, CKT, and AST) and the heart proteins (Troponin-I and -T) levels of the CMZ-group relative to control rats. These values were substantially ameliorated with both HSD and LT4 treatments.

**TABLE 2:** Effect of HSD and LT4 on the serum thyroid hormones level, the body weight changes, and the cardiac function biomarkers of CMZ-induced hypothyroidism in rats.

Parameter	Group			
	NC	CMZ	CMZ + HSD	CMZ + LT4
Total T3 (ng/dL)	89.32 ± 0.29^ab^	80.54 ± 1.24^a^	90.24 ± 4.39^b^	87.88 ± 0.51^ab^
Total T4 (µg/dL)	5.85 ± 0.06^b^	2.15 ± 0.24^a^	5.39 ± 0.07^b^	5.65 ± 0.12^b^
Free T3 (pg/mL)	5.44 ± 0.48^b^	2.68 ± 0.20^a^	5.57 ± 0.36^b^	4.62 ± 0.49^b^
Free T4 (ng/dL)	3.50 ± 0.12^c^	2.35 ± 0.15^a^	3.21 ± 0.08^bc^	2.88 ± 0.15^b^
TSH (µIU/mL)	0.01 ± 0.00^a^	0.06 ± 0.01^b^	0.02 ± 0.01^a^	0.03 ± 0.01^a^
Body weight (g)	283.78 ± 3.66^a^	312.57 ± 4.80 ^b^	275.08 ± 3.20^a^	276.23 ± 3.45^a^
CK-MB (U/L)	100.33 ± 3.51^a^	124.33 ± 3.75^b^	107.500 ± 2.61^a^	104.83 ± 5.02^a^
CKT (U/L)	250.67 ± 13.15^a^	444.67 ± 36.84^b^	280.67 ± 23.57^a^	300.50 ± 20.27^a^
AST (U/L)	102.00 ± 3.18^a^	147.50 ± 8.28^b^	109.67 ± 3.35^a^	121.00 ± 5.03^a^
Troponin-I (ng/mL)	0.36 ± 0.02^a^	2.04 ± 0.08^c^	0.55 ± 0.02^ab^	0.63 ± 0.06^b^
Troponin-T (pg/mL)	18.86 ± 0.96^a^	85.05 ± 5.87^b^	27.32 ± 2.23^a^	31.02 ± 2.28^a^

Data are mean ± SEM (*n* = 6). Different letters indicate significant differences (*p* < 0.05). Abbreviations: NC, normal control rats; CMZ, carbimazole-induced hypothyroid rats; CMZ + HSD, hypothyroid rats treated with hesperidin; CMZ + LT4, hypothyroid rats treated with levothyroxine; T3, triiodothyronine; T4, tetraiodothyronine; TSH, thyroid stimulating hormone; CK-MB, creatine kinase (muscle/blood); CKT, total creatine kinase; AST, aspartate aminotransferase.

### 3.2 Effect of HSD and LT4 on the serum lipid profile, cardiovascular risk indices, and cardiac redox status of CMZ-induced hypothyroidism

**FIGURE 1:**
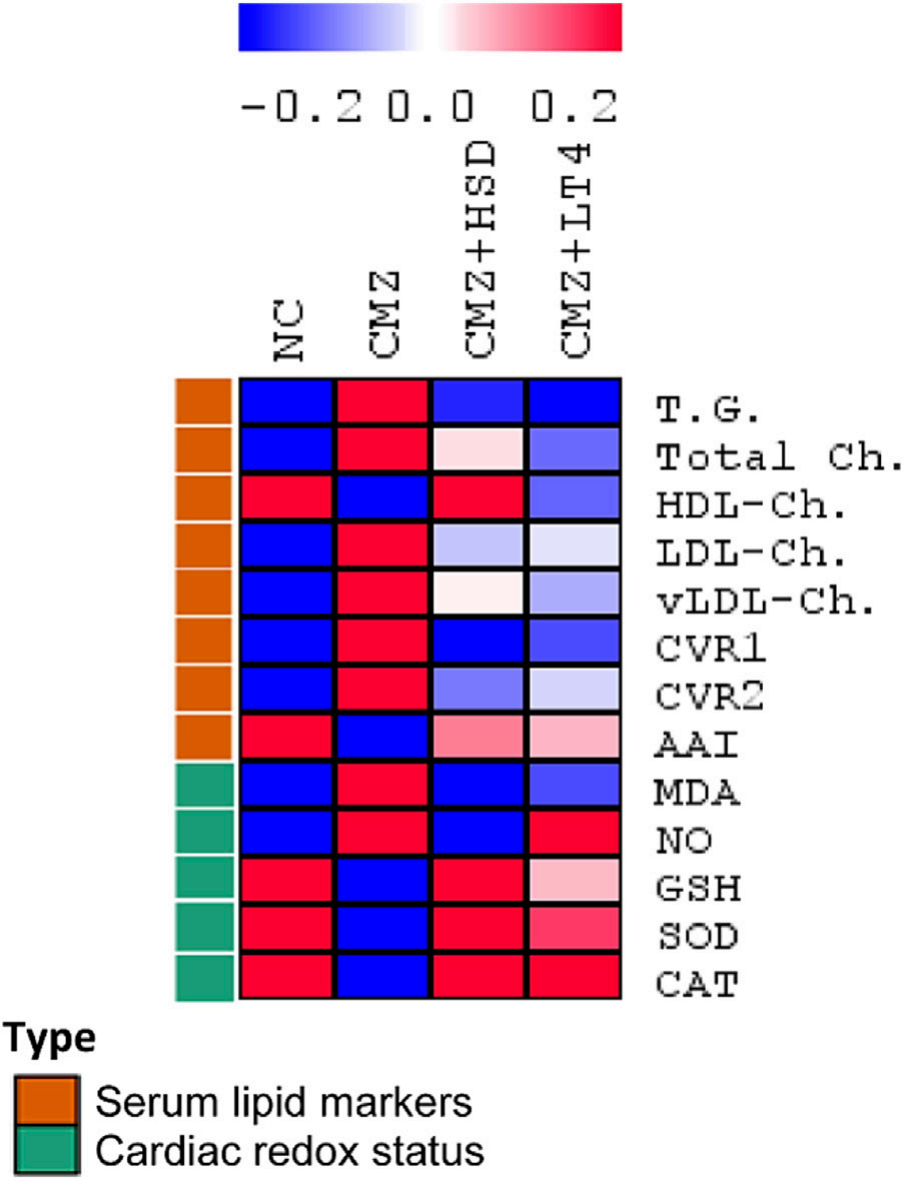
Effect of HSD and LT4 on the serum lipid profile, cardiovascular risk indices, and cardiac redox status of CMZ-induced hypothyroidism in rats. Data were normalized to remove variations in absolute values (Supplementary Table S1) and presented in a heat map to show the pattern across the different groups (*n* = 6). Abbreviations: NC, normal control rats; CMZ, carbimazole-induced hypothyroid rats; CMZ + HSD, hypothyroid rats treated with hesperidin; CMZ + LT4, hypothyroid rats treated with levothyroxine; T.G., triglycerides; Total Ch., total cholesterol; HDL-Ch., high density lipoprotein cholesterol; LDL-Ch., low density lipoprotein cholesterol; vLDL-Ch., very low density lipoprotein cholesterol; CVR1, cardiovascular risk index 1; CVR2, cardiovascular risk index 2; AAI, anti-atherogenic index; MDA, malondialdehyde; NO, nitric oxide; GSH, reduced glutathione; SOD, superoxide dismutase; CAT, catalase.

Progression of dyslipidemia is common in hypothyroidism (Delitala et al., 2017). Consistently, serum concentrations of TG, total Ch. and vLDL-Ch. were increased by about 1.5-fold each, while the LDL-Ch. level was 3-fold higher in the hypothyroid rats as compared to the control ones. In contrast, HDL-Ch. concentration was about 25% lower than the normal control group (Figure 1; Supplementary Table S1). These changes revealed a high risk of CVDs in the CMZ-group as confirmed by the elevated values of CVR1 and CVR2, and the depletion in AAI. Both HSD and LT4 administration induced potent hypolipidemic effects, while HSD was more effective in increasing HDL-Ch. levels than LT4 (Figure 1; Supplementary Table S1).

As hypothyroidism interferes with cardiac redox homeostasis and contributes to cardiovascular dysfunction (Baghcheghi et al., 2022), we set out to evaluate the heart’s redox status of the different experimental groups to compare the therapeutic potentials of HSD against LT4. Cardiac MDA and NO contents were significantly elevated by 3.6-fold and 2.9-fold, respectively, secondary to oral CMZ-intake in comparison to the NC-group. Moreover, cardiac GSH content and the antioxidant enzymatic activities of SOD and CAT were depleted by about 65%–70% each (Figure 1; Supplementary Table S1), revealing a severe oxidative stress condition. On the other hand, HSD and LT4 administration almost neutralized the negative impact of CMZ by reducing the stress markers (MDA and NO) and potentiating the antioxidant defenses. HSD showed a potent cardioprotective effect than LT4 (Figure 1; Supplementary Table S1).

### 3.3 Effect of HSD and LT4 on the cardiac pro- and anti-inflammatory mediators of CMZ-induced hypothyroidism

Hypothyroidism is a disorder that is commonly associated with elevations in the circulating inflammatory biomarkers (Lai et al., 2023), however, its effect on cardiac inflammation and the role of HSD and LT4 intake were not investigated yet. Our study revealed a marked increase in the pro-inflammatory cytokines TNF-α, IL-1β, IL-6, and IL-17 in the heart tissues of CMZ-hypothyroid rats. This was also associated with a significant decrease in the concentrations of IL-10, IL-37, and PPAR-α anti-inflammatory mediators (as compared to the NC-group; Table 3), uncovering serious cardiac inflammation. Consistent with the anti-inflammatory properties of HSD (Parhiz et al., 2015; Tejada et al., 2018), its oral intake substantially reduced the level of the pro-inflammatory cytokines by about 55%–70% relative to the CMZ-group. This immunomodulatory effect of HSD was more potent than the action of LT4 (Table 3). Both HSD and LT4 showed a comparable effect in restoring the concentration of the anti-inflammatory markers near the control values.

**TABLE 3:** Effect of HSD and LT4 on the cardiac pro- and anti-inflammatory mediators of CMZ-induced hypothyroidism in rats.

Parameter	Group			
	NC	CMZ	CMZ + HSD	CMZ + LT4
Pro-inflammatory mediators				
TNF-α (pg/mL)	13.53 ± 1.07^a^	92.70 ± 3.11^d^	26.74 ± 1.68^b^	39.43 ± 2.63^c^
IL-1β (pg/dL)	36.23 ± 1.44^a^	131.17 ± 4.08^c^	46.87 ± 3.71^ab^	56.28 ± 4.23 ^b^
IL-6 (pg/mL)	78.97 ± 2.68^a^	250.08 ± 4.39^d^	100.87 ± 1.16^b^	113.32 ± 3.34^c^
IL-17 (pg/dL)	56.62 ± 2.29^a^	176.07 ± 5.61^d^	79.20 ± 2.67^b^	97.12 ± 1.57^c^
Anti-inflammatory mediators				
IL-10 (pg/mL)	289.10 ± 12.16^c^	113.83 ± 2.04^a^	216.50 ± 2.08^b^	202.80 ± 2.85^b^
IL-37 (pg/mL)	126.00 ± 5.17^c^	52.48 ± 3.29^a^	110.77 ± 1.97^b^	99.97 ± 2.44^b^
PPAR-α (ng/mL)	9.73 ± 0.49^c^	2.53 ± 0.13^a^	7.92 ± 0.46^b^	6.82 ± 0.43^b^

Data are mean ± SEM (*n* = 6). Different letters indicate significant differences (*p* < 0.05). Abbreviations: NC, normal control rats; CMZ, carbimazole-induced hypothyroid rats; CMZ + HSD, hypothyroid rats treated with hesperidin; CMZ + LT4, hypothyroid rats treated with levothyroxine; TNF-α, tumor necrosis factor-alpha; IL, interleukin; PPAR-α, peroxisome proliferator activated receptor alpha.

### 3.4 Effect of HSD and LT4 on the cardiac miRNAs-92a, −499, and −21, and gene expression levels of NOS (endothelial and inducible) and caspase-3/BAX apoptotic markers of CMZ-induced hypothyroidism

**FIGURE 2:**
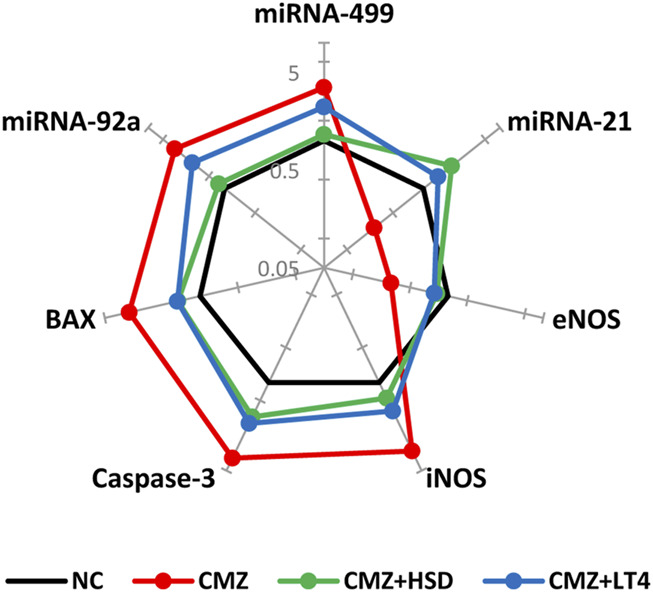
Effect of HSD and LT4 on the cardiac gene expression level of miRNAs-92a, -499, and -21, NOS (endothelial and inducible), and caspase-3/BAX apoptotic markers of CMZ-induced hypothyroidism in rats. Data were normalized corresponding to the mean values of the controls (*n* = 6). Absolute values in Supplementary Table S2. Abbreviations: NC, normal control rats; CMZ, carbimazole-induced hypothyroid rats; CMZ + HSD, hypothyroid rats treated with hesperidin; CMZ + LT4, hypothyroid rats treated with levothyroxine; miRNA, microRNA; eNOS, endothelial nitric oxide synthase; iNOS, inducible nitric oxide synthase; BAX, BCL2-associated X protein.

Ample evidence showed that THs signaling affects miRNAs expression (Zhang et al., 2017; Aranda, 2021). Therefore, it is crucial to explore the interdependence between hypothyroidism and cardiac miRNAs expression and the regulatory role of HSD in comparison to the LT4 replacement therapy. Our results showed elevated expression levels of miRNAs-92a and −499 in cardiomyocytes of hypothyroid rats as compared to NC-ones, while miRNA-21 showed an opposite pattern reflecting endothelial and cardiac dysfunction in CMZ-group (van Rooij et al., 2009; Chen et al., 2015; Aranda, 2021) (Figure 2; Supplementary Table S2). HSD intake alleviated these transcriptional changes up to the normal level more effectively than LT4.

Excessive NO production in cardiomyocytes of CMZ-hypothyroid rats directed us to investigate the expression level of both eNOS and iNOS. We found marked upregulation of iNOS with a downregulation of eNOS genes in the heart tissue of the CMZ-group relative to the normal controls, which suggests cardiovascular dysfunction. Both treatments restored their expression patterns (Figure 2; Supplementary Table S2) that might sustain the proper biological effect of NO on the cardiovascular system.

Detected oxidative and inflammatory disturbances in the cardiac muscles of the CMZ-group could render cardiomyocytes vulnerable to apoptosis. Elevated expression levels of apoptotic markers (caspase-3 and BAX) were apparent in the hypothyroid rats (Figure 2; Supplementary Table S2). In contrast, HSD and LT4 intakes seemed to attenuate this apoptotic behavior and control these transcript levels.

### 3.5 Effect of HSD and LT4 on the cardiac protein levels of Nrf2 and NF-κB of CMZ-induced hypothyroidism

**FIGURE 3:**
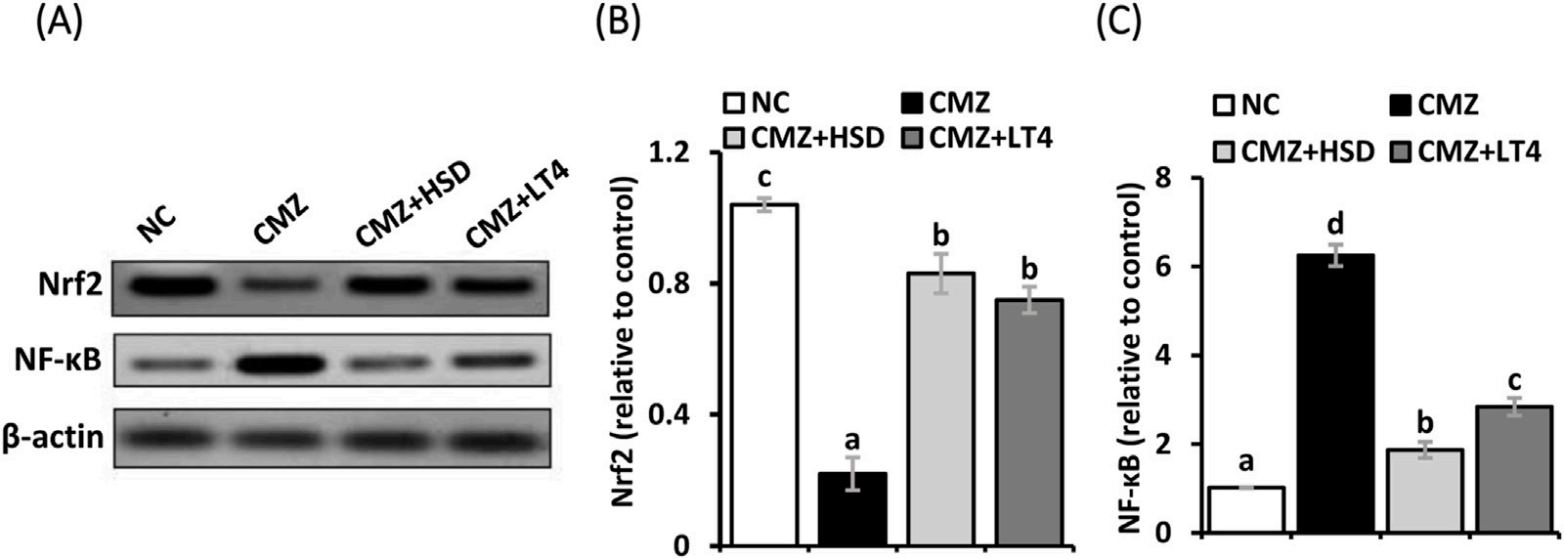
**(A–C)** Effect of HSD and LT4 on the cardiac protein expression level of Nrf2 and NF-κB of CMZ-induced hypothyroidism in rats. Data are mean ± SEM (*n* = 6). Abbreviations: NC, normal control rats; CMZ, carbimazole-induced hypothyroid rats; CMZ + HSD, hypothyroid rats treated with hesperidin; CMZ + LT4, hypothyroid rats treated with levothyroxine; Nrf2, nuclear factor erythroid 2-related factor 2; NF-κB, nuclear factor kappa B.

To understand the molecular basis underlying the detected oxidative stress and inflammation in the heart tissues of hypothyroid rats and to evaluate the therapeutic potency of HSD relative to LT4, we investigated the cardiac protein expression level of Nrf2 and NF-κB as key regulators of the antioxidant biomarkers and the inflammatory mediators, respectively. Figure 3 illustrates a significant depletion in the Nrf2 protein level in the cardiomyocytes of the CMZ-group while that of NF-κB was markedly elevated in comparison to the healthy controls. HSD and LT4 administration reestablished the protein level of Nrf2, while HSD was more effective in alleviating NF-κB expression level than LT4.

### 3.6 Effect of HSD and LT4 on the cardiac histopathology, ultrastructure, and immunoexpression of Gal-3 and MMP-9 of CMZ-induced hypothyroidism

**FIGURE 4:**
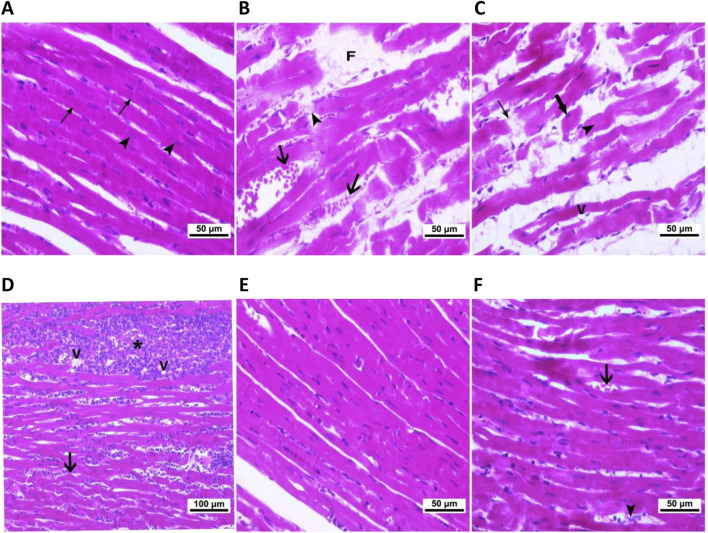
Cardiac histopathological alterations caused by CMZ-induced hypothyroidism and the effect of HSD and LT4 treatments (H&E staining). **(A)** Cardiomyocytes of the control group showing the normal intactness of the cardiac muscle fibers with proper striations (thin arrows) and regular vesicular nuclei (arrowhead). **(B–D)** Heart tissues of the CMZ-hypothyroid rats. **(B)** Showing myocytes losing striations (arrowhead), with congested blood vessels (arrows) and focal necrotic areas (F), **(C)** showing loss of continuity with adjacent myocytes (thin arrows) and myocytes losing their nuclei (arrowhead), fragmented cardiomyocytes with pyknotic nuclei (thick arrow), and vacuolation (V), and **(D)** showing irregular muscle fibers (arrow) and extensive inflammatory cellular infiltrations (star) associated with vacuolations (V). **(E)** Revealing potent improvement of the myocardial fibers after HSD administration. **(F)** Showing congested blood vessels (thick arrow) and inflammatory cellular infiltration (arrowhead) that are still apparent in the cardiomyocytes of LT4-treated rats. The histopathological scores are indicated in Table 4.

We used light and electron microscopy to examine the heart tissue sections from each experimental group and to assess the immunolocalization of the myocardial injury markers, Gal-3 and MMP-9, to thoroughly investigate the impact of hypothyroidism on cardiac tissues and the potential role of both treating agents. As compared to the typical cardiac histology of normal control rats (Figure 4A), the heart tissues of CMZ-treated rats showed congested blood vessels and focal necrotic areas (Figure 4B), fragmented cardiomyocytes with pyknotic nuclei and numerous vacuolations (Figure 4C), and irregular muscle fibers with extensive inflammatory cellular infiltrations (Figure 4D). In contrast, HSD administration almost kept the normal cardiac tissue configuration (Figure 4E; Table 4), while inflammatory cellular infiltration and congested blood vessels were still detected in the heart tissues of LT4 treated rats (Figure 4F; Table 4).

**TABLE 4:** Effect of CMZ-induced hypothyroidism and its treatment with HSD or LT4 on the cardiac histopathology and ultrastructural abnormalities.

Changes/Abnormalities	Group			
	NC	CMZ	CMZ + HSD	CMZ + LT4
Histopathological changes				
Fragmented cardiomyocytes	−	++	−/+	−/+
pyknotic nuclei	−/+	+	+	−/+
Vacuolations	−/+	+	−/+	−/+
Necrotic areas	-	++	−/+	+
Congested blood vessels	−/+	++	−/+	+
Inflammatory cellular infiltrations	-	++	−/+	+
Ultrastructural abnormalities				
Fragmented/degenerated myofibrils	−	++	−/+	+
Abnormal mitochondria	−	++	−/+	−/+
Shrunken nuclei	−	+	−	−/+
Swelling of SER	−	+	−/+	−/+
Vacuolations	−/+	++	−/+	+

(−) absent, (−/+) few “≤ 10%”, (+) moderate, and (++) severe (*n* = 3). Abbreviations: NC, normal control rats; CMZ, carbimazole-induced hypothyroid rats; CMZ + HSD, hypothyroid rats treated with hesperidin; CMZ + LT4, hypothyroid rats treated with levothyroxine.

**FIGURE 5:**
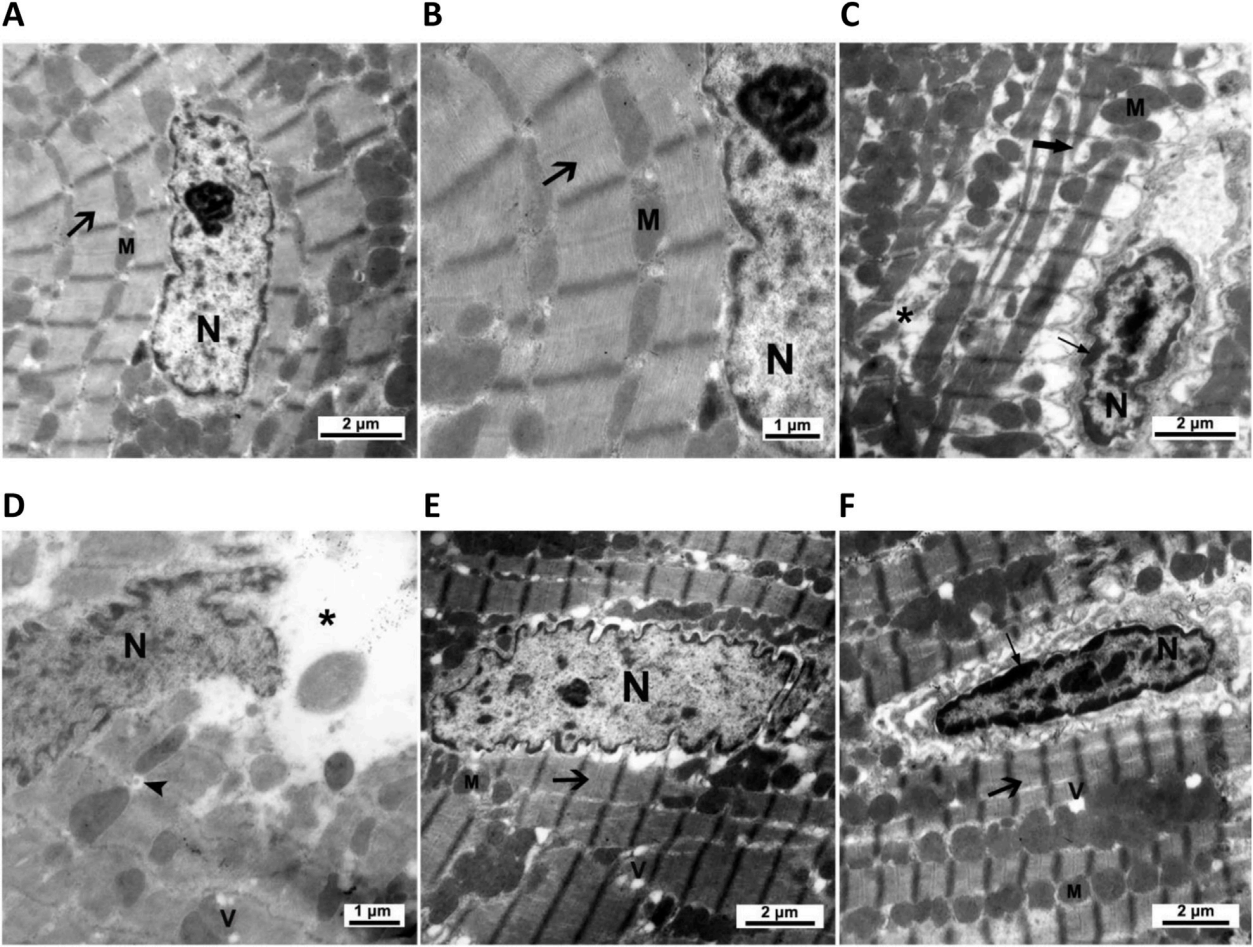
Cardiac ultrastructural abnormalities caused by CMZ-induced hypothyroidism and the effect of HSD and LT4 treatments. **(A,B)** Cardiomyocytes of the control group showing regular striations of the myofibrils (arrow), a normal shape of mitochondria (M), and a proper distribution of chromatin in the nucleus (N). **(C,D)** Cardiomyocytes of the CMZ-hypothyroid rats revealing fragmented (thick arrow) and degenerated (star) myofibrils, irregular aggregations of electron-dense mitochondria (M) with ill-defined cristae, shrunken nucleus (N) with dense clumps of heterochromatin (thin arrow), swelling of smooth endoplasmic reticulum (arrowhead), and appearance of vacuoles (V). **(E)** Cardiomyocytes of the HSD-treated group showing marked amelioration of the myofibrillar striations (arrow), regular shape of mitochondria (M), and euchromatic nucleus (N) with few cytoplasmic vacuolations (V). **(F)** Cardiomyocytes of the LT4-treated group showing improvement in the myofibrillar striations (thick arrow) and mitochondria (M), while the nucleus has dense clumps of heterochromatin (thin arrow) with few vacuoles in the sarcoplasm (V). Ultrastructural abnormality scores are in Table 4.

The ultrastructural examination of the control cardiomyocytes showed regular myofibrillar striations, normal mitochondrial shape, and proper nuclear chromatin distribution (Figures 5A,B). Cardiomyocytes of CMZ-treated rats revealed fragmented/degenerated myofibrils, electron-dense mitochondria with ill-defined cristae, shrunken nuclei with dense clumps of heterochromatin, cytoplasmic vacuolations, and swelling of smooth endoplasmic reticulum (Figures 5C,D). Cardiomyocytes of HSD-treated rats appeared more structured (Figure 5E) with fewer abnormalities than those detected under LT4 treatment where the nuclei had electron-dense heterochromatin, and few vacuoles were noticed in the sarcoplasm (Figure 5F; Table 4).

**FIGURE 6:**
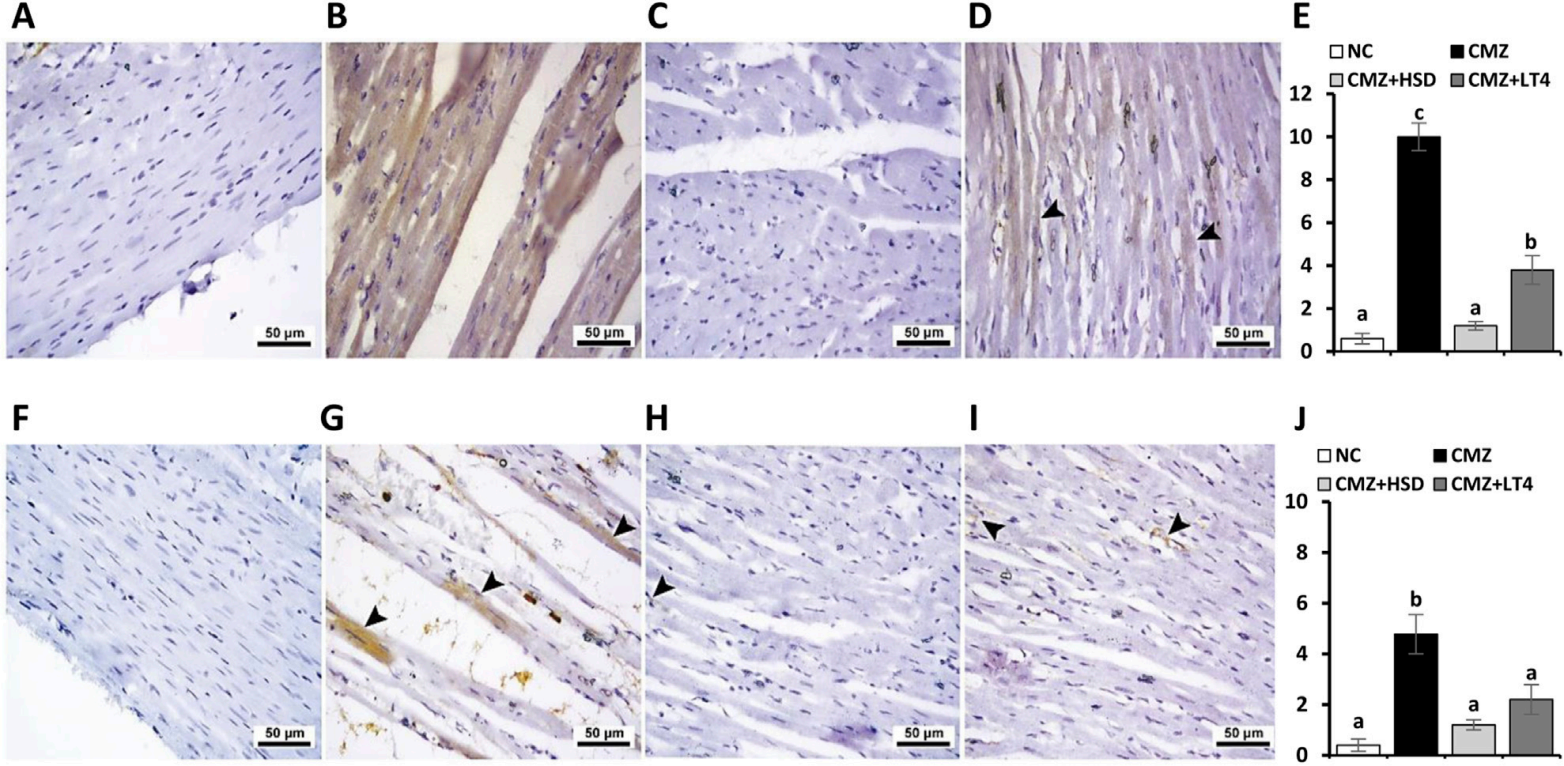
Effect of CMZ-induced hypothyroidism and its treatment with HSD or LT4 on the cardiac immunoexpression of **(A–E)** Gal-3, **(F–J)** MMP-9, and their quantification using H-score. **(A)** Cardiac tissue of the normal control group showing negative immunostaining toward Gal-3, **(B)** strong brown immunopositive reactivity of Gal-3 in the cardiomyocytes of CMZ-hypothyroid rats, **(C)** absence of reactivity for Gal-3 in cardiac myofibers of the HSD-treated group, while **(D)** showing mild cytoplasmic immune expression of Gal-3 in the cardiac tissues of the LT4-treated group (arrowheads). **(F)** Negative immunoreactivity of MMP-9 in the cardiomyocytes of the control group, **(G)** significant immune expression of MMP-9 that is localized in the damaged areas of the cardiomyocytes of the CMZ-hypothyroid group (arrowheads), **(H)** myofibers from the HSD-treated rats with very weak MMP-9 expression (arrowhead), and **(I)** revealing faint MMP-9 staining in the cardiac tissue fibers of the LT4-treated group (arrowheads).

Regarding the immune expression analysis of Gal-3 and MMP-9, and as compared to controls (Figures 6A, F, respectively), both were strongly localized in the heart tissues of the hypothyroid rats (Figures 6B, E, G, J, respectively). In contrast, the HSD-treated group showed either negative or very weak immunoreactivity toward Gal-3 and MMP-9 (Figures 6C,E,H,J, respectively), while their expression under LT4 administration ranged from faint to mild (Figures 6D,E,I,J, respectively). Therefore, we can conclude that the treatment with HSD caused marked protection from the myocardium damage compared to the LT4 effect.

## 4 Discussion

Our data revealed a significant decrease in THs level (total T3, total T4, free T3, and free T4) with a subsequent increase in serum TSH after oral intake of CMZ as compared to NC-rats. Hypothyroidism has been implicated in potentiating cardiac injury (Jabbar et al., 2017). Therefore, we set out to evaluate the heart function biomarkers to explore the possible therapeutic benefits of HSD. The serum cardiac enzymes activities of CK-MB, CKT, and AST, as well as the heart protein levels of troponin-I and -T, were substantially increased in CMZ-induced hypothyroid rats. Concisely, hypothyroidism has been linked to a higher risk of ischemic heart disease (Kannan et al., 2018). This ischemia impairs Ca^2+^ channels of the sarcoplasmic reticulum (SR), resulting in Ca^2+^ over-release to the cytosol (Zucchi and Ronca-Testoni, 1997). Tappia et al. (2001) have reported the association between cytosolic Ca^2+^ overload and oxidative stress which exposes myocardial cells to damage and the sarcolemma to hyperpermeability, leading to leakage of cardiac enzymes and proteins into the bloodstream. In addition, CMZ itself has been extensively reported to have toxic effects on different organs (Greene and Morgan, 1956; Bitrus et al., 2018; Hussein Naser et al., 2022). It alters the antioxidant defense system, causing oxidative stress at a toxic level that impairs the cell membrane functional integrity and exacerbates the seep of cardiac enzymes out of the sarcoplasm. Oral intake of HSD showed a remarkable amelioration in these altered parameters. This cardioprotective activity of HSD may be due to its Ca^2+^ channel blocking property (Selvaraj and Pugalendi, 2010), and to its antioxidant and ROS scavenging actions (Welbat et al., 2020).

Hypothyroidism is directly correlated with dyslipidemia; the major risk factor of coronary diseases and atherosclerosis (Delitala et al., 2017). Our data revealed a significant increase in the circulating lipid markers (TG, total Ch., LDL-Ch., and vLDL-Ch.) and CVR indices of CMZ-treated rats, while HDL-Ch. and AAI were significantly decreased as compared to controls. Hypercholesterolemia in hypothyroidism is mainly due to the reduction in low-density lipoprotein receptor (LDLR) expression that suppresses hepatic cholesterol uptake from the circulation (Liu and Peng, 2022). Also, low levels of THs slow down the rate of intrahepatic cholesterol secretion into the bile, reducing cholesterol catabolism/turnover and accounting for the appearance of dyslipidemia (Liu and Peng, 2022). The increase in serum TG level in hypothyroidism usually refers to the decrease in TG-rich lipoprotein clearance secondary to inhibition of lipoprotein lipase activity (Liu and Peng, 2022). HSD administration possibly protects from dyslipidemia through the downregulation of fatty acid synthase (FAS) and acetyl coenzyme A carboxylase alpha (ACCα), and upregulation of ATP-binding cassette transporters G8 (ABCG8) protein expressions in the liver (Sun et al., 2017). FAS and ACCα are two key enzymes in fatty acids and TG synthesis, while ABCG8 plays a vital role in the cholesterol efflux into the bile.

Detected hypothyroidism and dyslipidemia seem to expose the heart tissue to oxidative stress (Yamakawa et al., 2021; Baghcheghi et al., 2022). CMZ-treated rats showed elevated levels of cardiac MDA and NO, with a marked decrease in GSH content, and CAT and SOD activities. Rather than the previously described toxic effect of CMZ, Torun et al. (2009) have reported the association between hypothyroidism and dysfunction of the mitochondrial respiratory chain that aggravates free radical production and attenuates the antioxidant defenses. Further, Diekman et al. (1998) found that LDL-Ch. from hypothyroid patients is more vulnerable to oxidation, forming oxidative LDL-Ch. (oxLDL) that ensues high oxidative stress levels. This study showed that the heart tissues of HSD-treated rats are better protected from oxidative stress than those treated with LT4. HSD boosted the antioxidant enzymes activities and decreased the intracellular pro-oxidants secondary to activation of Nrf2 signaling. Nrf2 is a master inducible factor for a large network of cytoprotective and antioxidant enzymes (Li et al., 2008). A recent study by Huang et al. (2024) showed that HSD activates Nrf2 by interfering with the Nrf2–Keap1 interaction. Keap1 is the protein sequestering Nrf2 in the cytoplasm. The interfering action of HSD causes conformational changes in Keap1 cysteine residues, resulting in Nrf2 dissociation and translocation to the nucleus. Nrf2, then, binds to the antioxidant response elements (ARE) and activates the expression of various antioxidant genes. In line with Elavarasan et al. (2012), our data revealed an elevated protein expression level of Nrf2 in cardiomyocytes of HSD-treated rats. The stabilized redox status in the HSD-treated group might illustrate the sarcolemma integrity and the decrease in the circulating levels of heart function enzymes and proteins.

The biological action of NO on the cardiovascular system depends on the NOS isoform that is activated. It appears that NO produced by iNOS promotes inflammation and cardiomyocyte apoptosis, while that produced by eNOS is an important vasodilator and protects from cardiac apoptosis (Schulz et al., 2005). In healthy cardiomyocytes, eNOS is the most expressed isoform, while iNOS is usually undetectable (Vanhoutte et al., 2016). Therefore, we set out to explore the gene expression level of iNOS and eNOS in cardiac tissues of the different experimental groups. We found a substantial upregulation of iNOS with a downregulation of eNOS genes in the cardiac tissues of hypothyroid rats as compared to the controls, which suggests cardiovascular dysfunction. Induction of iNOS expression is mediated through activation of the transcription factor nuclear factor-kappa B (NF-κB) (Kang et al., 2011). Accordingly, the current study revealed a significant increase in NF-κB protein expression level in the heart tissues of the CMZ-group. Further, Cai et al. (2015) reported that T3 enhances the transcription of the eNOS gene and, consequently, its deficiency suppresses eNOS expression and contributes to cardiac dysfunction progression. On the other hand, the transcript level of iNOS in HSD and LT4-treated rats was significantly decreased relative to CMZ-ones. This is possibly due to the potent depletion in NF-κB protein expression level (Parhiz et al., 2015). Upregulation of eNOS in both treated groups might refer to their ability to sustain the circulating level of total T3, as well as activation of Akt protein kinase B which can directly phosphorylate eNOS and stimulate its action (Chiou et al., 2008; Vanhoutte et al., 2016).

MiRNAs are newly discovered gene regulators that have been linked to diverse biological activities. An earlier study on cardiac muscles demonstrated the crosstalk between miRNAs and THs signaling, shedding light on the role of miRNAs in modulating THs functionality (Zhang et al., 2017). The miRNAs analysis revealed changed expression levels of miRNAs-92a, −499, and −21 in the heart tissues of hypothyroid rats as compared to the control ones. MiRNAs-92a and −499 were upregulated, while miRNA-21 was downregulated. MiRNA-92a is a crucial miRNA that disrupts endothelial functions in response to oxidative stress. Briefly, accumulation of oxidized lipids in hypothyroidism imposes endothelial cells (ECs) to oxidative stress and activates the sterol regulatory element-binding protein-2 (SREBP2) transactivation of miRNA-92a that initiates endothelial innate immunity, producing proinflammatory cytokines/chemokines that impair eNOS-derived NO bioavailability, a key feature of endothelial dysfunction (Chen et al., 2015). MiRNA-499 is a cardiac-specific miRNA that induces cardiomyocyte remodeling in response to stress (van Rooij et al., 2009; Montgomery et al., 2011). It upregulates the expression of myosin heavy chain-7 (MyHC7) which is closely associated with cardiac hypertrophy, cardiomyopathy, and acute myocardial infarction. According to Van Rooij et al. (2009), hypothyroidism induces miRNA-499 expression in adults’ hearts and results in a higher level of MyHC7 expression, causing cardiac dysfunction. In contrast, miRNA-21 is essential for thyroid hormone synthesis, and its deregulation (as in the CMZ-group) is associated with the loss of THs bioavailability and the subsequent HF progression. MiRNA-21 has been shown to target the degradation of type-3 deiodinase (DIO3) mRNA. DIO3 is a member of the selenoenzymes family which catabolizes T4 and T3 and terminates their action (Aranda, 2021). The expression profile of these miRNAs was better controlled in the HSD-treated group than the LT4-treated one. To our knowledge, the mechanistic regulation of miRNAs by HSD is still unknown. However, it has been described that polyphenols such as curcumin, resveratrol, and ellagic acid can regulate key transcription factors like p53 and c-myc that can bind to miRNA promoter elements and control miRNA expression. Taking this into consideration, it could be suggested that HSD (as a polyphenolic compound) may modulate miRNA expression in a similar way (Milenkovic et al., 2013). Therefore, we thought that HSD restored eNOS expression by inhibiting miRNA-92a, kept cardiomyocytes’ integrity by suppressing miRNA-499, and maintained THs functionality by inducing miRNA-21 expression. Accordingly, the blockade of miRNAs-92a and −499, and the upregulation of miRNA-21 seem like a novel therapeutic strategy to protect from CMZ-induced hypothyroidism and cardiac dysfunction in rats.

Clinical and experimental studies showed an interplay between hypothyroidism and inflammation (Zhou et al., 2018; Lai et al., 2023). However, their co-influence on myocardium has not been thoroughly investigated. Our results demonstrate a significant increase in TNF-α, IL-1β, IL-6, and IL-17 proinflammatory markers in the cardiac tissues of CMZ-induced hypothyroid rats. TNF-α is a master mediator of inflammation in the pathogenesis of many diseases, including thyroid dysfunction. Thyroid infiltrating lymphocytes (TIL) in hypothyroidism is a reliable source of TNF-α (Zhang et al., 2022). Intriguingly, the heart tissues contain resident macrophages which produce TNF-α in response to changes in systemic THs homeostasis (Wenzek et al., 2022; Chen et al., 2024). TNF-α alters Ca^2+^ influx and release by SR, mediates induction of iNOS, stimulates cardiomyocyte apoptosis by activating MAPK/JNK-signaling, and interacts with the TNF-receptor associated death domain that activates the endonucleases to destroy the cellular DNA (Ferrari, 1999; Rolski and Blyszczuk, 2020). IL-1β is a highly inflammatory cytokine that is produced by macrophages in disease states. Elevated serum levels of IL-1β are one of the main characteristic features of hypothyroidism (Mikos et al., 2014). It decreases DIO1 activity (that catalyzes T4 conversion into T3) and represses TR expression through the NF-κB/activator protein (AP)-1 dependent pathway (Kwakkel et al., 2007). Interestingly, members of the IL-1 family are present constitutively in all healthy mesenchymal cells, including the myocardium (Abbate et al., 2020). IL-1β precursor is activated into a mature cytokine by triggering the caspase/NLRP3-inflammasome axis secondary to intracellular or extracellular danger-associated signals such as hypercholesterolemia (Rajamaki et al., 2010); a detected feature of hypothyroidism. This dyslipidemia seems to activate IL-1β production in the cardiomyocytes. IL-1β in cardiac tissues induces oxidative stress, activates coagulation factors, contributes to atherosclerotic plaque formation, and directly modulates cardiac contractility as being a cardio-depressant factor that leads to circulatory collapse and shocks (Abbate et al., 2020). High TSH levels, in response to hypothyroidism, induce the release of IL-6 via NF-κB activation (Antunes et al., 2008). IL-6 reduces DIO1 activity and further decreases THs production (Enia et al., 2007). In cardiomyocytes, IL-6 positively regulates JAK/STAT signaling that mediates cardiac inflammation, induces C-reactive protein production and leukocytic infiltration, and aggravates mitochondrial dysfunction and oxidative stress (Su et al., 2021). IL-17 has been recognized as a crucial contributor to the pathogenesis of hypothyroidism (Lai et al., 2023). It is produced by the thyroid follicular cells themselves and TIL. In cardiac tissues, IL-17 induces the expression of diverse proinflammatory cytokines/chemokines and demonstrates strong synergic action with IL-1β and TNF-α to induce an inflammatory milieu that augments cardiac disease progression through different pathways (Allam et al., 2018). In contrast, IL-10 and IL-37 are strong natural suppressors of inflammation. Hypothyroidism is closely associated with perturbation in IL-10 and IL-37 (Majnaric et al., 2022; Lai et al., 2023). The heart tissues of CMZ-induced hypothyroid rats showed a marked decrease in IL-10 and IL-37 concentrations. IL-10 is a key anti-inflammatory mediator that is secreted by cardiac macrophages and other cells of both the myeloid and lymphoid lineages (Saraiva et al., 2020). It blocks signaling cascades induced by the master pro-inflammatory switches NF-κB and TNF-α (Driessler et al., 2004). IL-37 is a strategic anti-inflammatory mediator that belongs to the IL-1 family (Majnaric et al., 2022). It potentially activates the anti-inflammatory pathways PTEN and STAT3. It also inhibits the major proinflammatory IKK- and MAPK-dependent pathways, TLRs signaling, and NLRP3 inflammasome. Further, IL-37 can abolish effects of the incendiary cytokines (TNF-α, IL-1, and IL-6) and translocate to the nucleus to suppress the transcription of their genes (Majnaric et al., 2022). Therefore, neutralizing the proinflammatory cytokines and stimulating the anti-inflammatory ones might subdue the adverse cardiovascular events in hypothyroidism. Oral intake of HSD substantially decreased cardiac levels of TNF-α, IL-1β, IL-6, and IL-17 cytokines than LT4. Several studies have reported the immunomodulatory properties of HSD (Parhiz et al., 2015; Tejada et al., 2018). In line with our findings, the anti-inflammatory effect of HSD may be mediated through the attenuation of NF-κB signaling that orchestrates a variety of inflammatory cytokines (Parhiz et al., 2015). Of note, HSD represses the NF-κB pathway by blocking the activity of the IκB kinase (IKK). IKK is an enzymatic complex that phosphorylates the inhibitor of κB member α (IκBα). This blocking activity of HSD prevents the nuclear translocation of NF-κB subunits (mainly p65/RelA), suppressing the transcription of various inflammatory genes (Kang et al., 2011; Gao et al., 2021). Studies by Parhiz et al. (2015) and Tejada et al. (2018) have illustrated further molecular action of HSD by modulating MAPK/JNK signaling and the extracellular signal-regulated kinase (ERK). Treatment with HSD enhanced the secretion of IL-10 in the cardiomyocytes (Parhiz et al., 2015). To our knowledge, this is the first report that elucidates HSD’s capability to induce IL-37 production in cardiac tissues. This provides another instance of supporting the immunomodulatory properties of HSD.

PPAR-α is a transcriptional factor that belongs to the nuclear receptor superfamily. It shares structural similarities with a subfamily of receptors that includes TR and controls the transcription of target genes through heterodimerization with RXR. PPARs and TR crosstalk via multiple mechanisms to affect diverse biological functions, including lipid metabolism, where their signaling pathways are coupled (Kouidhi and Clerget-Froidevaux, 2018). PPAR-α is predominantly expressed in the heart tissues, and its activation lowers the circulating lipids and reduces their store level (Pawlak et al., 2015). As well, PPAR-α negatively regulates the pro-inflammatory mediators by inhibiting NF-κB signaling (Pawlak et al., 2015). Xiong et al. (2019) previously demonstrated that HSD upregulates PPAR-α expression in the adipose and liver tissues. This study is the first to document a significant increase in PPAR-α levels in the heart tissues of HSD-treated rats, suggesting further protection from hypothyroidism-related dyslipidemia and cardiac inflammation. Interestingly, PPAR-α is one of the T3-regulated genes, and the activation of PPAR-α may restore TR expression and THs functionality (Lu and Cheng, 2010). Therefore, TR and PPAR-α can reciprocally affect each other’s activity. Accordingly, we suppose that PPAR-α upregulation in HSD-treated rats might participate in restoring THs levels.

In association with the declined antioxidants and the elevated inflammation in the cardiac muscles of CMZ-induced hypothyroidism, cardiomyocytes are susceptible to death. Here, CMZ intake induced cardiac apoptosis by upregulating the gene expression levels of BAX and caspase-3. Bax increases the mitochondrial membrane permeability, causing the release of cytochrome c which activates the programmed cell death by inducing caspase-3 action (Varisli et al., 2022). Levels of BAX and caspase-3 expression were nearly normalized by HSD. This anti-apoptotic action might be attributed to HSD’s anti-inflammatory and antioxidant properties (Parhiz et al., 2015; Varisli et al., 2022). The cardioprotective efficacy of HSD is consistent with our histological and cytological observations.

In this study, CMZ-induced hypothyroidism subjected the heart tissues to various degenerative lesions and even led to necrosis and apoptotic cardiac cell death. This might explain the leakage of cardiac enzymes and proteins into the serum. Similar histopathological and ultrastructural abnormalities were detected by Nepomnyashchikh et al. (2013) and Ayuob et al. (2016) in response to hypothyroidism. Further, the immune expression of Gal-3 and MMP-9 (as markers of cardiac inflammation and fibrosis) was significantly elevated in the heart tissues of the CMZ-group. Gal-3 is a β-galactoside-binding protein of the lectin family that is involved in regulating cell-matrix interactions and tissue repair (Zaborska et al., 2023). The expression of Gal-3 in the heart tissues is usually low, and its prolonged overexpression has been related to the pathophysiology of heart failure (HF) (Zaborska et al., 2023). At the injury site, Gal-3 is secreted by injured and inflammatory cells and activates quiescent myofibroblasts to produce cytoskeleton proteins through TGF-β-dependent and independent pathways leading to inappropriate remodeling of the damaged tissue and the development of fibrosis (Zaborska et al., 2023). MMP-9 is a zinc-dependent endopeptidase that is involved in the remodeling and degradation of the extracellular matrix (ECM) and plays a key role in biological and pathophysiological processes (Morishita et al., 2017). Abnormalities in MMP-9 expression result in the progression of various inflammatory diseases and contribute to the pathogenesis of HF (Morishita et al., 2017). MMP-9 is mainly secreted by neutrophils, macrophages, and fibroblasts. Infiltration of these cells into the heart tissues overexpresses MMP-9 causing degradation of the connective tissues and ECM, triggering cardiac pathogenesis and exacerbating the immune cells migration to the inflammatory sites (Al-Roub et al., 2023). Both inflammatory cytokines and redox biomarkers boost Gal-3 and MMP-9 production (Bittner et al., 2010; Fort-Gallifa et al., 2017; Al-Roub et al., 2023). Therefore, suppressing inflammation and activating the antioxidant defenses are important to inhibit Gal-3 and MMP-9 actions and prevent cardiac tissue damage. Our study revealed a marked amelioration in the myocardium of HSD-treated rats compared to CMZ and relative to the LT4 effect. This might refer to HSD’s anti-inflammatory, antioxidant, and antiapoptotic properties (Parhiz et al., 2015; Varisli et al., 2022), as well as the role of HSD in Gal-3 antagonism (Odun-Ayo and Reddy, 2021) and MMP-9 inhibition (Lee et al., 2018).

Further mechanistic studies could be conducted to better understand the dual thyroprotective and cardioprotective action of HSD. These could involve exploring HSD’s impact on the PI3K/Akt/mTOR pathway, which plays an important role in thyroid cell survival and functionality (Kenessey and Ojamaa et al., 2006), and the AMPK pathway, which regulates energy metabolism, reduces lipid accumulation in arteries, and helps prevent atherosclerosis (Heidary Moghaddam et al., 2021). HSD appears to offer a range of therapeutic benefits with a good safety profile in animal studies (Li et al., 2019). The current study investigated the action of HSD after 9 weeks of administration at daily doses of 200 mg/kg b.wt. (Varışlı et al., 2022). Throughout the experiment, this dose did not induce any physiological or behavioral abnormalities that require investigation. To our knowledge, there is a lack of data describing potential long-term adverse effects of HSD on the thyroid and cardiovascular health. Longer-term studies are recommended to reveal whether HSD consistently protects the thyroid and cardiovascular systems over extended periods. Our findings provide valuable insight into how HSD works to improve hypothyroidism-related cardiac dysfunction in rats, with promising results that provide a basis for considering HSD in early-stage clinical trials. However, further studies such as additional animal trials, safety tests, and pharmacokinetic data would be necessary before proceeding to clinical investigation.

## 5 Conclusion

**FIGURE 7:**
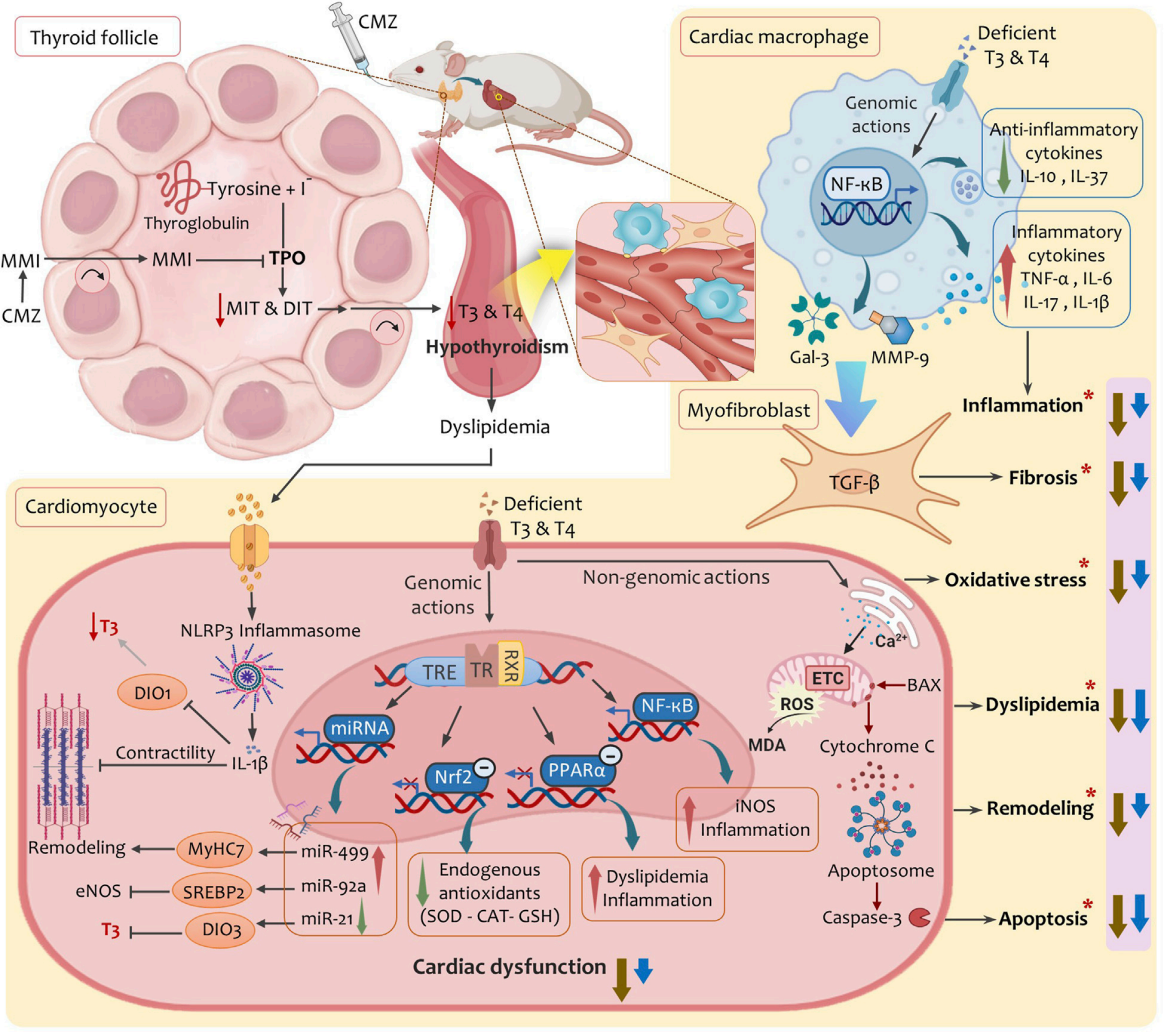
A proposed mechanistic model summarises the multimodal effects of HSD in alleviating hypothyroidism-related cardiac dysfunction. Abbreviations: BAX, BCL2-associated X protein; CAT, catalase; CMZ, carbimazole; DIO1, deiodinase type-1; DIO3, deiodinase type-3; DIT, Diiodothyronine; eNOS, endothelial nitric oxide synthase; ETC, electron transport chain; Gal-3, galectin-3; GSH, glutathione; IL, interleukin; iNOS, inducible nitric oxide synthase; MDA, malondialdehyde; MiRNA, microRNA; MIT, monoiodothyronine; MMI, methimazole; MMP-9, matrix metalloproteinase-9; MyHC7, myosin heavy chain-7; NF-κB, nuclear factor kappa-B; Nrf2, nuclear factor erythroid 2-related factor 2; PPAR-α, peroxisome proliferator activated receptor alpha; ROS, reactive oxygen species; RXR, retinoid-X-receptor; SOD, superoxide dismutase; SREBP2, sterol regulatory element-binding protein-2; TGF-β, Transforming growth factor beta; TPO, thyroid peroxidase; TR, thyroid hormone receptor; TRE, thyroid hormone response element; (*), factors lead to cardiac dysfunction in hypothyroidism; Brown arrows, effects of hesperidin; Blue arrows: effects of levothyroxine.

In conclusion, HSD provided dual thyroprotective and cardioprotective effects against CMZ-induced hypothyroidism and subsequent cardiac dysfunction. Our study showed an integrative action of HSD on multiple control points (Figure 7). It induced potent hypolipidemic, anti-inflammatory, and anti-apoptotic effects, restored cardiac redox homeostasis, kept the myocardium integrity, and modulated cardiac miRNAs levels. This collectively alleviated THs bioavailability and functionality in the cardiovascular system and protected from hypothyroidism-related cardiac dysfunction.

## Data Availability

The original contributions presented in the study are included in the article/Supplementary Material, further inquiries can be directed to the corresponding author.
