# Supplementary material for: Hesperidin alleviates hypothyroidism-related cardiac dysfunction by targeting cardiac miRNAs, Nrf2/NF-κB signaling, oxidative stress and inflammation
Source: Front Pharmacol. 2025 Jul 2;16:1553992. doi: 10.3389/fphar.2025.1553992 (PMC12263625; doi:10.3389/fphar.2025.1553992)
Supplement: Supplementary file 1 [file Table1.docx]

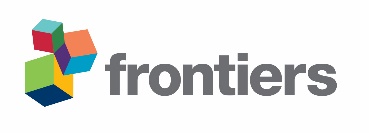


***Supplementary Material***

| Parameter | Group | | | |
| --- | --- | --- | --- | --- |
|  | NC | CMZ | CMZ+HSD | CMZ+LT4 |
| Triglycerides (mg/dL) | 71.5 ± 2.74^a^ | 108 ± 1.71^b^ | 82.25 ± 5.13^a^ | 80.83 ±3.44^a^ |
| Total Cholesterol (mg/dL) | 66 ± 1.26^a^ | 92.67 ± 2.13^c^ | 78 ± 0.47^b^ | 76.50 ± 3.38^b^ |
| HDL-Cholesterol (mg/dL) | 46.16 ± 4.1^b^ | 35.13 ± 0.99^a^ | 45.77 ± 1.78^b^ | 41.17 ± 3.46^ab^ |
| LDL-Cholesterol (mg/dL) | 10.8 ± 3.31^a^ | 35.93 ± 2.95^c^ | 18.95 ± 1.43^b^ | 19.17± 1.64^b^ |
| vLDL-Cholesterol (mg/dL) | 12.6 ± 0.26^a^ | 21.60 ± 0.34^c^ | 16.45 ± 1.03^b^ | 16.17± 0.69^b^ |
| CVR1 | 1.51 ± 0.05^a^ | 2.65 ± 0.13^c^ | 1.83 ± 0.02^b^ | 1.86 ± 0.05^b^ |
| CVR2 | 0.24 ± 0.06^a^ | 1.04 ± 0.12^b^ | 0.45 ± 0.04^a^ | 0.47± 0.04^a^ |
| AAI | 197.32 ± 20.87^c^ | 61.96 ± 3.90^a^ | 120.66 ± 3.31^b^ | 118.21 ± 7.22^b^ |
| MDA (nmol/g tissue) | 45.27 ± 4.06^a^ | 165.75 ± 4.95^c^ | 62.55 ± 2.20^b^ | 70.15 ± 5.16^b^ |
| NO (nmol/mg protein) | 1.33 ± 0.04^a^ | 3.80 ±0.17^c^ | 1.40 ± 0.08^a^ | 2.23 ± 0.08^b^ |
| GSH (mg/g tissue) | 100.16 ± 5.53^c^ | 33.95 ± 3.69^a^ | 79.48 ± 4.83^b^ | 66.95 ± 4.30 ^b^ |
| SOD (U/g tissue) | 25.05 ± 1.77^c^ | 8.80 ± 0.44^a^ | 21.75 ± 1.19^bc^ | 18.53 ± 0.90^b^ |
| CAT (U/g tissue) | 175.97 ± 5.09^c^ | 49.83 ± 3.37^a^ | 137.95 ± 3.53^b^ | 128.85 ± 2.5^b^ |

**Supplemental Table S1.** Effect of HSD and LT4 on the serum lipid profile, cardiovascular risk indices, and cardiac redox status of CMZ-induced hypothyroidism in rats.

Data are mean ± SEM (*n =* 6). Different letters indicate significant differences (*p* < 0.05). Abbreviations: NC, normal control rats; CMZ, carbimazole-induced hypothyroid rats; CMZ+HSD, hypothyroid rats treated with hesperidin; CMZ+LT4, hypothyroid rats treated with levothyroxine; CVR1, cardiovascular risk index 1; CVR2, cardiovascular risk index 2; AAI, anti-atherogenic index; MDA, malondialdehyde; NO, nitric oxide; GSH, reduced glutathione; SOD, superoxide dismutase; CAT, catalase.

| Parameter | Group | | | |
| --- | --- | --- | --- | --- |
|  | NC | CMZ | CMZ+HSD | CMZ+LT4 |
| miRNA-92a | 1.09 ± 0.02^a^ | 4.88 ± 0.108^d^ | 1.30 ± 0.06^a^ | 2.88 ± 0.10^b^ |
| miRNA-499 | 1.09 ± 0.04^a^ | 3.81 ± 0.04^d^ | 1.26 ± 0.02^b^ | 2.41 ± 0.06^c^ |
| miRNA-21 | 1.01 ± 0.05^b^ | 0.23 ± 0.02^a^ | 2.37 ± 0.05^d^ | 1.571 ± 0.04^c^ |
| eNOS | 1.03 ± 0.01^c^ | 0.26 ± 0.04^a^ | 0.79 ± 0.03^b^ | 0.74 ± 0.04^b^ |
| iNOS | 1.03 ± 0.01^a^ | 6.17 ± 0.67^b^ | 1.55 ± 0.16^a^ | 2.17 ± 0.19^a^ |
| Caspase-3 | 1.03 ± 0.02^a^ | 7.42 ± 0.66^c^ | 2.53 ± 0.52^b^ | 3.00 ± 0.13^b^ |
| BAX | 1.02 ± 0.01^a^ | 5.64 ± 0.36^b^ | 1.74 ± 0.11^a^ | 1.76 ± 0.22^a^ |

**Supplemental Table S2.** Effect of HSD and LT4 on the cardiac gene expression level of miRNAs-92a, -499, and -21, NOS (endothelial and inducible), and caspase-3/BAX apoptotic markers of CMZ-induced hypothyroidism in rats.

Data are mean ± SEM (*n* = 6) and normalized relative to control. Different letters indicate significant differences (*p* < 0.05). Abbreviations: NC, normal control rats; CMZ, carbimazole-induced hypothyroid rats; CMZ+HSD, hypothyroid rats treated with hesperidin; CMZ+LT4, hypothyroid rats treated with levothyroxine; eNOS, endothelial nitric oxide synthase; iNOS, inducible nitric oxide synthase; BAX, BCL2-associated X protein.
